# A novel Python module for statistical analysis of turbulence (P-SAT) in geophysical flows

**DOI:** 10.1038/s41598-021-83212-1

**Published:** 2021-02-17

**Authors:** Mayank Agarwal, Vishal Deshpande, David Katoshevski, Bimlesh Kumar

**Affiliations:** 1 Department of Computer Science and Engineering, Indian Institute of Technology Patna, Patna, India; 2 Department of Civil and Environmental Engineering, Indian Institute of Technology Patna, Patna, India; 3 Department of Civil and Environmental Engineering, Ben-Gurion University of the Negev, Beer-Sheva, Israel; 4 Department of Civil Engineering, Indian Institute of Technology Guwahati, Guwahati, India

**Keywords:** Hydrology, Computer science, Civil engineering

## Abstract

We present Python Statistical Analysis of Turbulence (P-SAT), a lightweight, Python framework that can automate the process of parsing, filtering, computation of various turbulent statistics, spectra computation for steady flows. P-SAT framework is capable to work with single as well as on batch inputs. The framework quickly filters the raw velocity data using various methods like velocity correlation, signal-to-noise ratio (SNR), and acceleration thresholding method in order to de-spike the velocity signal of steady flows. It is flexible enough to provide default threshold values in methods like correlation, SNR, acceleration thresholding and also provide the end user with an option to provide a user defined value. The framework generates a ***.csv*** file at the end of the execution, which contains various turbulent parameters mentioned earlier. The P-SAT framework can handle velocity time series of steady flows as well as unsteady flows. The P-SAT framework is capable to obtain mean velocities from instantaneous velocities of unsteady flows by using Fourier-component based averaging method. Since P-SAT framework is developed using Python, it can be deployed and executed across the widely used operating systems. The GitHub link for the P-SAT framework is: https://github.com/mayank265/flume.git.

## Introduction

We have developed Python-Statistical Analysis of Turbulence (P-SAT), an open-source, lightweight Python framework that can de-spike (identify spikes and replace them) the raw velocity time series data obtained from an acoustic Doppler velocimeter (ADV) device using various filtering methods. The P-SAT framework also computes a range of turbulent statistics like mean velocities, variance, skewness, kurtosis, Reynolds stresses, third order correlations, 2D as well as 3D fluxes of turbulent kinetic energy, turbulent kinetic energy dissipation, conditional statistics of quadrants, octants and their corresponding probabilities, spectrum of a given signal etc. The P-SAT framework also exports all the turbulent statistics into a ***.csv*** so that it can be used in future for analysis.

Authors of the present study believe that the P-SAT framework would be of a significant help to those researchers who are associated with research involving statistical analyses of the turbulent flows in steady as well as in unsteady flow environments. The motivation behind development of the P-SAT framework was the authors’ experience on working with various third party tools and utilities that are commercially available in the scientific community. The authors found few major issues that led to the development of P-SAT framework : (1) To the best of our knowledge, there is no single tool which can handle instantaneous velocity signals of steady flows and unsteady flows together, and is able to compute majority of the turbulent statistics that the P-SAT framework computes.

As working with the turbulent flows involves complex computations (such as: calculating mean velocities in study/unsteady flow environments, Reynolds shear stress (RSS) calculations, turbulent kinetic energy (TKE) calculations, finding third order moments of velocity fluctuations, TKE dissipation, calculations of contributions towards total RSS production from different quadrants, octant analysis and determination of the octant probabilities, calculations of spectral density functions of velocities etc.), it becomes cumbersome to compute these quantities in $Microsoft \ Excel$ or write custom scripts for the same. (2) Support for batch processing of files: Experiments performed on a laboratory flume often consists of multiple readings at multiple points thus requiring a tool to work on batch of input files. (3) Commercial tools are expensive to use and are often closed source. Also, the flexibility provided to the end users is often limited.

The P-SAT framework is capable of handling all of the above issues. It provides a rich turbulent statistics for the given set of input files. It is an open source tool developed in Python with the source code available on GitHub for the scientific community to use it and modify it. We have chosen Python for developing P-SAT because Python has been widely adopted in the recent years because of the availability of various libraries and extensive community support. Many modules for varying applications like modeling, analysis, and optimization of electric power systems^1^, SciPy^2^, Gene Ontology analyses^3^, ice sheet models^4^, machine learning^5^, data logger for coupled fluid-structure simulations^6^, have used Python exclusively and made available to the scientific community.

**Figure 1:**
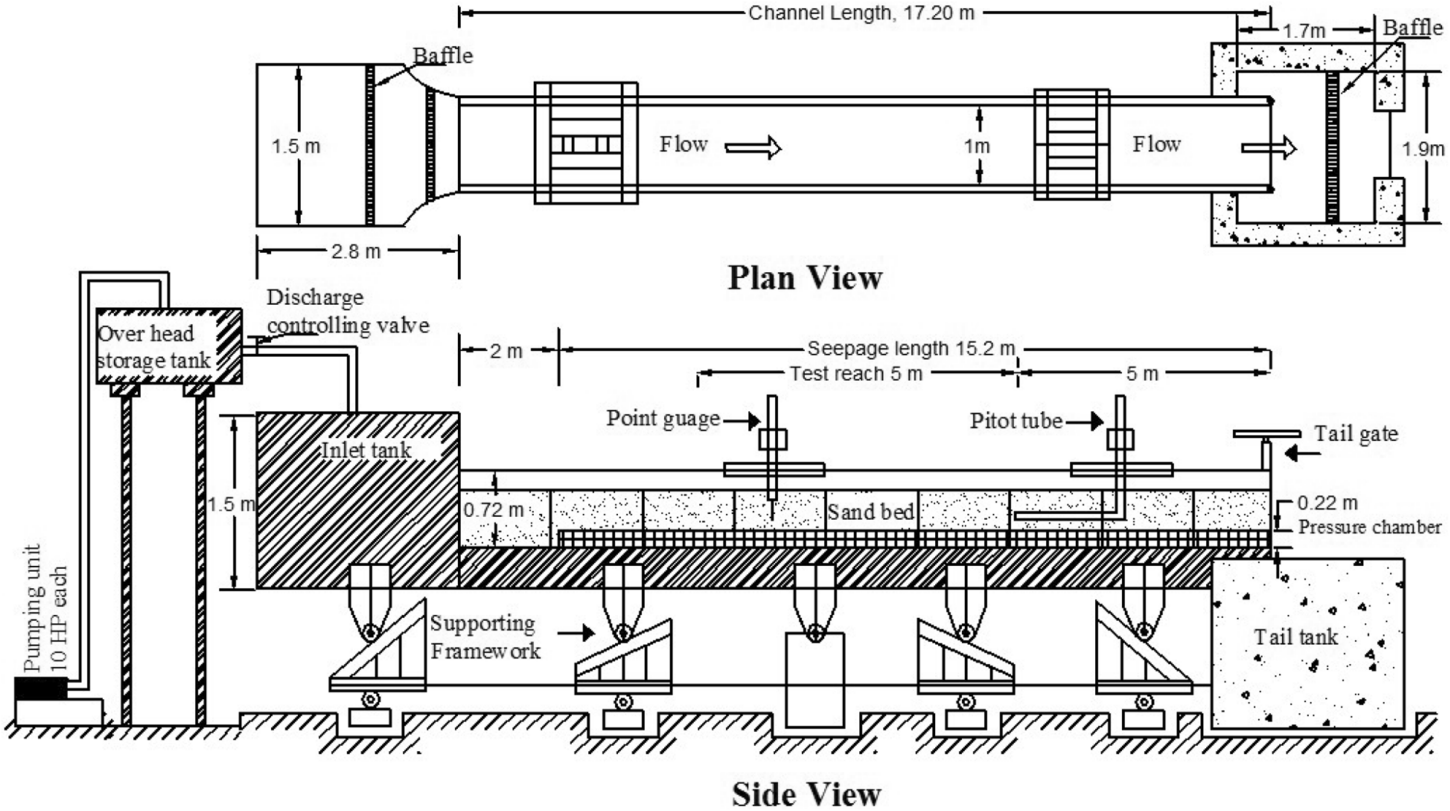
Schematic diagram of the experimental flume.

The authors have been studying fluvial geomorphology and transport behavior of sediments (erosion/deposition) in alluvial channels, where turbulence plays as a key parameter in various fluvial environments such as: flow in a channel with curvilinear cross-section^7^, flows over bedforms^8^, flows with vegetation^9^, flows around bridge piers^10^, and flows in sand mined channels^11^. For the present study, we carried out extensive experiments in a laboratory flume setup at Department of Civil Engineering, Indian Institute of Technology Guwahati. The experimental setup is shown in Fig. 1 and more details regarding the experiments and measurements are explained in Sect. 3. Instantaneous velocity readings along the vertical plane were taken using a four-beam, down-looking, Vectrino+ acoustic Doppler velocimeter (ADV) probe manufactured by Nortek. Measurements were carried out on multiple heights, and at each height, 30,000 samples were collected for 5 min (i.e., sampling rate of 100 Hz).

ADVs demonstrate a proven technology for capturing 3D velocity signals in environmental flows e.g.,^12–15^. However, the raw velocity signals captured by ADVs may contain spikes caused by aliasing of the Doppler signal. Another issue with the raw signal maybe the Doppler noise floor also. To clean the velocity record from the previously mentioned issues, post-processing of the raw velocity signal may be necessary. These spikes have an adverse effect on the turbulent statistics that are computed and hence, they must be removed from the time series data collected by the ADV. The process of removal of spikes is known as de-spiking and there are various approaches for the same^16^. The P-SAT framework implements three de-spiking approaches: velocity correlation, SNR and acceleration thresholding method.

The paper is organized as follows: Sect. 2 consists of definitions of various statistical parameters of turbulence for the steady flow. It also provides an insight into the computation of the mean velocity component from the instantaneous velocities for highly unsteady flows. Section 3 describes the experimental setup in details and provides the various measurements involved. The P-SAT framework is described in Sect. 4. It details out all the requirements for the P-SAT framework, the data format, execution steps, and the parameters computed by the P-SAT framework. Section 5 provides an insight into the discussion about the P-SAT framework. Section 6 concludes the paper and provides a possible future course of work for the P-SAT framework. We also include an appendix section where more information about the individual Python files that are used along with the files that are created by the P-SAT framework are described. This would be useful for the developers who wish to enhance the P-SAT framework and add features to it.

**Table 1:** List of symbols.

${{f}_{cutoff}}$	Cut-off frequency
${{f}_{TKEu}}$	Flux of the turbulent kinetic energy in streamwise direction
${{f}_{TKEw}}$	Flux of the turbulent kinetic energy in vertical direction
$2DF_{TKEu}$	(Non-dimensional) 2D Flux of the turbulent kinetic energy in streamwise direction
$2Df_{TKEu}$	2D flux of the turbulent kinetic energy in streamwise direction
$2DF_{TKEw}$	(Non-dimensional) $2Df_{TKEw}$
$2Df_{TKEw}$	2D flux of the turbulent kinetic energy in vertical direction
$3DF_{TKEu}$	(Non-dimensional) $3Df_{TKEu}$
$3Df_{TKEu}$	3D flux of the turbulent kinetic energy in streamwise direction
$3DF_{TKEw}$	(Non-dimensional) $3Df_{TKEw}$
$3Df_{TKEw}$	3D flux of the turbulent kinetic energy in vertical direction
$d_{50}$	Median particle diameter
${\delta }_{i,H}$	Indicator function for quadrant analysis
*e*	Dissipation of the turbulent kinetic energy
*ED*	(Non-dimensional) *e*
*g*	Gravitational constant
*h*	Flow depth
*H*	Hole size
*k*	Fourier component
$\lambda _a$	Parameter for acceleration thresholding method
$M_{30}, M_{03} \, M_{12}, M_{21}$	Third order moments of velocity
*n*	Number of samples
*Q*	Flow discharge
*Re*	Reynolds number
${{\rho }_{water}}$	Density of water
$S_{i,H}$	Fractional contribution towards RSS production
${{\sigma }_{g}}$	Gradation coefficient for sand
${\sigma }_{u}, {\sigma }_{v}, {\sigma }_{w},$	Standard deviation of velocity in the streamwise, lateral, and vertical directions, respectively
${{\tau }_{uw}}, {{\tau }_{uv}},$ and ${{\tau }_{vw}}$	Reynolds shear stresses
*T*	Time period of measurement
$u', v', w'$	Fluctuating components of streamwise, lateral and vertical velocity, respectively
*U*, *V*, *W*	Time-averaged streamwise, lateral and vertical velocity, resp
${{U}_{uf}}$	Instantaneous velocities
$U_*$	Shear velocity
$\overline{U_{uf}}$	Mean velocity component for unsteady flow
$U_i, V_i, W_i$	Instantaneous streamwise, lateral and vertical velocity, resp.
${{\varphi }^{o}}$	Angle of repose

## Theoretical foundations

This section has been divided into two subsections: In Sect. 2.1, definitions of various statistical parameters of turbulence for the steady flow are presented. In Sect. 2.2, calculation of the mean velocity component from the instantaneous velocities for highly unsteady flow is presented.

### Definitions of various statistical parameters of turbulence for steady flow

Since the tool calculates several statistical parameters from raw data, it is better if these statistical parameters are defined first. All the notation relating to the statistical parameters are depicted in Table 1. Time-averaged streamwise (*U*), lateral (*V*), and vertical (*W*) velocities have been calculated as:

1 $$\begin{aligned}&U=\frac{1}{n}\sum \limits _{i=1}^{n}{{{U}_{i}}} \end{aligned}$$

2 $$\begin{aligned}&V=\frac{1}{n}\sum \limits _{i=1}^{n}{{{V}_{i}}} \end{aligned}$$

3 $$\begin{aligned}&W=\frac{1}{n}\sum \limits _{i=1}^{n}{{{W}_{i}}} \end{aligned}$$

where, $U_i, V_i,$ and $W_i$ are the instantaneous velocities in the streamwise, lateral and vertical directions, respectively, and *n* is the number of samples taken. Velocity variance for all three components of the velocity can be given by:

4 $$\begin{aligned}&u\_var=\overline{u{{'}^{2}}}=\frac{1}{n}\sum \limits _{i=1}^{n}{u{{'}^{2}}} \end{aligned}$$

5 $$\begin{aligned}&v\_var=\overline{v{{'}^{2}}}=\frac{1}{n}\sum \limits _{i=1}^{n}{v{{'}^{2}}} \end{aligned}$$

6 $$\begin{aligned}&w\_var=\overline{w{{'}^{2}}}=\frac{1}{n}\sum \limits _{i=1}^{n}{w{{'}^{2}}} \end{aligned}$$

where $u', v'$ and $w'$ are the fluctuating components of velocities in the streamwise, lateral, and vertical directions, respectively. The square root of the velocity variance is the standard deviation. The shape of the probability density function is illustrated by its skewness. The skewness also depicts the relative contribution of positive and negative velocity fluctuations to the formation of the velocity pattern. Skewness for the velocities in all three directions can be given by:

7 $$\begin{aligned}&u\_skew={{\left( \overline{u{{'}^{2}}} \right) }^{{}^{-3}/{}_{2}}}\frac{1}{n}\sum \limits _{i=1}^{n}{u{{'}^{3}}} \end{aligned}$$

8 $$\begin{aligned}&v\_skew={{\left( \overline{v{{'}^{2}}} \right) }^{{}^{-3}/{}_{2}}}\frac{1}{n}\sum \limits _{i=1}^{n}{v{{'}^{3}}} \end{aligned}$$

9 $$\begin{aligned}&w\_skew={{\left( \overline{w{{'}^{2}}} \right) }^{{}^{-3}/{}_{2}}}\frac{1}{n}\sum \limits _{i=1}^{n}{w{{'}^{3}}} \end{aligned}$$

Kurtosis is a statistical measure that defines how heavily the tails of a distribution differ from the tails of a normal distribution. In other words, kurtosis identifies whether the tails of a given distribution contain extreme values. Kurtosis of the probability distribution of a real-valued random variable (3D velocity in the present case) is given by:

10 $$\begin{aligned}&u\_kurt={{\left( \overline{u{{'}^{2}}} \right) }^{-2}}\frac{1}{n}\sum \limits _{i=1}^{n}{u{{'}^{4}}} \end{aligned}$$

11 $$\begin{aligned}&v\_kurt={{\left( \overline{v{{'}^{2}}} \right) }^{-2}}\frac{1}{n}\sum \limits _{i=1}^{n}{v{{'}^{4}}} \end{aligned}$$

12 $$\begin{aligned}&w\_kurt={{\left( \overline{w{{'}^{2}}} \right) }^{-2}}\frac{1}{n}\sum \limits _{i=1}^{n}{w{{'}^{4}}} \end{aligned}$$

Reynolds stresses furnish very important information about the transfer of momentum in turbulent flows. Reynolds stresses are the components of a symmetric second order tensor where the diagonal components are called the Reynolds normal stresses (RNS) and the off diagonal components are called the Reynolds shear stresses (RSS)^17^. Reynolds shear stresses (${{\tau }_{uw}}, {{\tau }_{uv}},$ and ${{\tau }_{vw}}$) can be calculated as:

13 $$\begin{aligned}&{{\tau }_{uw}}=-{{\rho }_{water}}\overline{u'w'} \end{aligned}$$

14 $$\begin{aligned}&\overline{u'w'}=\frac{1}{n}\sum \limits _{i=1}^{n}{\left( {{U}_{i}}-U \right) \left( {{W}_{i}}-W \right) } \end{aligned}$$

15 $$\begin{aligned}&{{\tau }_{uv}}=-{{\rho }_{water}}\overline{u'v'} \end{aligned}$$

16 $$\begin{aligned}&\overline{u'v'}=\frac{1}{n}\sum \limits _{i=1}^{n}{\left( {{U}_{i}}-U \right) \left( {{V}_{i}}-V \right) } \end{aligned}$$

17 $$\begin{aligned}&{{\tau }_{vw}}=-{{\rho }_{water}}\overline{v'w'} \end{aligned}$$

18 $$\begin{aligned}&\overline{v'w'}=\frac{1}{n}\sum \limits _{i=1}^{n}{\left( {{V}_{i}}-V \right) \left( {{W}_{i}}-W \right) } \end{aligned}$$

where, ${{\rho }_{water}}$ is the density of water. The degree of flow anisotropy is measured by the ratio, ${\sigma }_{w}/{\sigma }_{u}$ and can be given by following expression:

19 $$\begin{aligned} Anisotropy=\frac{{{\sigma }_{w}}}{{{\sigma }_{u}}}=\frac{\sqrt{\overline{w'w'}}}{\sqrt{\overline{u'u'}}} \end{aligned}$$

where, ${\sigma }_{w}$ and ${\sigma }_{u}$ are the standard deviations of the vertical and streamwise velocities, respectively.

The third statistical moments or the skewness indicate non-symmetric distributions. Zero skewness indicate symmetric distribution about the mean or the Gaussian distribution, while negative and positive values of skewness show that the distribution is skewed towards left and right, respectively, around the mean. According to Raupach^18^, the third moments of velocity fluctuations, ${{M}_{jk}}=\overline{{{\widehat{u}}^{j}}{{\widehat{w}}^{k}}}, {\text {where}}j+k=3$, and ${\widehat{u}}=\frac{u'}{{{\left( \overline{u'u'} \right) }^{0.5}}}$, ${\widehat{w}}=\frac{w'}{{{\left( \overline{w'w'} \right) }^{0.5}}}$ can be expressed as:

20 $$\begin{aligned} {{M}_{30}}&=\frac{\overline{u{{'}^{3}}}}{{{\left( \overline{u'u'} \right) }^{1.5}}} \end{aligned}$$

21 $$\begin{aligned} {{M}_{03}}&=\frac{\overline{w{{'}^{3}}}}{{{\left( \overline{w'w'} \right) }^{1.5}}} \end{aligned}$$

22 $$\begin{aligned} {{M}_{21}}&=\frac{\overline{u{{'}^{2}}w'}}{\left( \overline{u'u'} \right) {{\left( \overline{w'w'} \right) }^{0.5}}} \end{aligned}$$

23 $$\begin{aligned} {{M}_{12}}&=\frac{\overline{u'w{{'}^{2}}}}{{{\left( \overline{u'u'} \right) }^{0.5}}\left( \overline{w'w'} \right) } \end{aligned}$$

The total Reynolds shear stress ($-\overline{u'w'}$) at any given point is the sum of different types of bursting events. Thus, depending upon the relative sign of instantaneous values of velocity fluctuations $u'$ and $w'$, the bursting events can be plotted in four different quadrants $(i = 1, 2, 3, 4)$ of the $(u', w')$ plane^19^ i.e., outward interactions $(i = 1: u'> 0, w' > 0)$, ejections $(i = 2: u' < 0, w' > 0)$, inward interactions$(i = 3: u'< 0, w' < 0)$, and sweeps$(i = 4: u' > 0, w' < 0)$. At any point in the flow field, the contribution to the total Reynolds shear stress through different ways of momentum transfer can be calculated as:

24 $$\begin{aligned} {{\left\langle u'w' \right\rangle }_{i,H}}=\underset{T\rightarrow 0}{\mathop {\lim }}\,\frac{1}{T}\int _{0}^{T}{u'\left( t \right) w'}\left( t \right) {{\delta }_{i,H}}\left[ u'\left( t \right), w'\left( t \right) \right] dt \end{aligned}$$

where angle brackets correspond to conditional averaging, *T* is the sampling time, and ${\delta }_{i,H}$ is the indicator function. The definition of the indicator function can be given as:

25 $$\begin{aligned} {{\delta }_{i,H}}\left[ u'\left( t \right) ,w'\left( t \right) \right] =\left\{ \begin{aligned}&1, \;{\text{if }}\left( u',w' \right) \;{\text{are in quadrant }}i\,{\text {and if }}\left| u'w' \right| \ge H{{\sigma }_{u}}{{\sigma }_{w}} \\&0, \;{\text{otherwise.}} \end{aligned} \right. \end{aligned}$$

where *H* is the parameter defined by the hyperbolic hole region^20^ which allows the investigation of larger contribution to the total Reynolds shear stress from various quadrants. Fractional contribution to the total Reynolds shear stress from different quadrants can be defined as:

26 $$\begin{aligned} {{S}_{i,H}}=\frac{{{\left\langle u'w' \right\rangle }_{i,H}}}{\overline{u'w'}} \end{aligned}$$

$S_{i,H}$ is negative for outward and inward interactions $(i = 1, 3)$ and is positive for ejections and sweeps $(i = 2, 4)$. Eq. (26) implies that for $H = 0$ when hole size disappears, sum of the fractional contributions from all the quadrants equals to unity ($\sum \limits _{i=1}^{4}{{{S}_{i,0}}}=1$).

Octant analysis is advantageous when the user wishes to analyze turbulent structures with a strong three dimensionality. Octant analysis is carried out by considering all three components of velocity fluctuations (Table 2).

**Table 2:** Determination of octant.

Octant name	$U'$	$V'$	$W'$
$-1$	${>}0$	${>}0$	${<}0$
$+1$	${>}0$	${>}0$	${>}0$
$-2$	${<}0$	${<}0$	${>}0$
$+2$	${<}0$	${>}0$	${>}0$
$-3$	${<}0$	${<}0$	${<}0$
$+3$	${<}0$	${>}0$	${<}0$
$-4$	${>}0$	${<}0$	${<}0$
$+4$	${>}0$	${>}0$	${<}0$

2D fluxes of the turbulent kinetic energy in the streamwise ($2D{{f}_{TKEu}}$) and vertical ($2D{{f}_{TKEw}}$) directions^18^ can be calculated as:

27 $$\begin{aligned}&2D{{f}_{TKEu}}=\frac{3}{4}\left( \overline{u{{'}^{3}}}+\overline{u'w{{'}^{2}}} \right) \end{aligned}$$

28 $$\begin{aligned}&2D{{f}_{TKEw}}=\frac{3}{4}\left( \overline{u{{'}^{2}}w'}+\overline{w{{'}^{3}}} \right) \end{aligned}$$

Further, these 2D turbulent kinetic energy fluxes have been made non-dimensional by dividing them by the shear velocity ($U_*$):

29 $$\begin{aligned}&2D{{F}_{TKEu}}={}^{2D{{f}_{TKEu}}}/{}_{{{U}_{*}}^{3}} \end{aligned}$$

30 $$\begin{aligned}&2D{{F}_{TKEw}}={}^{2D{{f}_{TKEw}}}/{}_{{{U}_{*}}^{3}} \end{aligned}$$

3D fluxes of the turbulent kinetic energy in the streamwise (${3D{f}_{TKEu}}$) and vertical ($3D{{f}_{TKEw}}$) directions can be calculated as:

31 $$\begin{aligned}&3D{{f}_{TKEu}}=\frac{1}{2}\left( \overline{u{{'}^{3}}}+\overline{u'v{{'}^{2}}}+\overline{u'w{{'}^{2}}} \right) \end{aligned}$$

32 $$\begin{aligned}&3D{{f}_{TKEw}}=\frac{1}{2}\left( \overline{u{{'}^{2}}w'}+\overline{v{{'}^{2}}w'}+\overline{w{{'}^{3}}} \right) \end{aligned}$$

Similarly, these 3D turbulent kinetic energy fluxes can be made non-dimensional by dividing them by the shear velocity ($U_*$):

33 $$\begin{aligned}&3D{{F}_{TKEu}}={}^{3D{{f}_{TKEu}}}/{}_{{{U}_{*}}^{3}} \end{aligned}$$

34 $$\begin{aligned}&3D{{F}_{TKEw}}={}^{3D{{f}_{TKEw}}}/{}_{{{U}_{*}}^{3}} \end{aligned}$$

The turbulent kinetic energy (TKE) is defined as half the sum of the variances of the velocity components and can be calculated as:

35 $$\begin{aligned} TKE=\frac{1}{2}\left( \overline{u'u'}+\overline{v'v'}+\overline{w'w'} \right) \end{aligned}$$

Dissipation of the turbulent kinetic energy (*e*) and its non-dimensional form (*ED*) can be calculated by the following expressions:

36 $$\begin{aligned}&{\text{Turbulent kinetic energy dissipation }}e=\frac{15\upsilon }{{{U}^{2}}}{{\left( \frac{\partial u'}{\partial t} \right) }^{2}} \end{aligned}$$

37 $$\begin{aligned}&ED=e\frac{h}{{{U}_{*}}^{3}} \end{aligned}$$

The P-SAT framework also allows user to compute the power spectra of filtered/unfiltered three-dimensional velocities by converting a time domain signal into frequency domain using a discrete Fast Fourier Transform (FFT). The spectrum of a filtered signal represents the mean square amplitude of that signal. In other words, spectrum shows the energy of a signal at any given frequency^21^. Energy in turbulence is received at large scales, and its dissipation occurs at small scales. Spectra allows us to think about the way, in which the energy is exchanged among eddies of different sizes.

### Calculation of the mean velocity component from the instantaneous velocities for highly unsteady flow

In order to analyze structure of the unsteady flows, determination of the mean velocity component $\overline{(U_{uf}}$) from the instantaneous velocities (${{U}_{uf}}$) is an essential parameter. From the definition point of view, instantaneous velocities of the unsteady flow can be decomposed into the mean velocities and fluctuating components in a following way:

38 $$\begin{aligned} {{U}_{uf}}\left( t \right) =\overline{{{U}_{uf}}}\left( t \right) \pm u' \end{aligned}$$

There are several methods available to find out the mean velocity component from the instantaneous velocities^22^. However, the Fourier-component method has been found most suitable for the determination of the mean velocity component in unsteady flows^23^. The determination of the mean velocities in unsteady flows using the Fourier-component method can be done as follows: Time dependent instantaneous velocities ${{U}_{ufi}}$ (where *i* = 1, 2, ..., *n*) are transformed into the frequency domain by using a discrete Fourier transform and only the frequency components lower than a cutoff frequency (${{f}_{cutoff}}$) are taken as the representative values of the mean velocities $\overline{(U_{ufi}}$) as follows:

39 $$\begin{aligned} {{f}_{cutoff}}&={}^{\left( k-1 \right) }/{}_{2T}.\end{aligned}$$

40 $$\begin{aligned} \overline{{{U}_{ufi}}}&=\frac{1}{2}{{a}_{0}}+\sum \limits _{j=1}^{\left( k-1 \right) /2\;}{\left( {{a}_{j}}\cos {{\omega }_{ij}}+{{b}_{j}}\sin {{\omega }_{ij}} \right) } \end{aligned}$$

where,

41 $$\begin{aligned} {{a}_{j}}&=\frac{2}{n}\sum \limits _{i=1}^{n}{{{U}_{i}}\cos {{\omega }_{ij}}} \end{aligned}$$

42 $$\begin{aligned} {{b}_{j}}&=\frac{2}{n}\sum \limits _{i=1}^{n}{{{U}_{i}}\sin {{\omega }_{ij}}} \end{aligned}$$

and

43 $$\begin{aligned} {{\omega }_{ij}}=2\pi j\left( \frac{i}{N} \right) \end{aligned}$$

for ${j} = 0, 1, 2, ..., ({k} - 1)/2$. Here, *n* is the number of samples collected in the time period (*T*) of a measurement. Nezu et al.^22^ have suggested to adopt the value of cutoff frequency (${{f}_{cutoff}}$) to be smaller than the burst frequency of turbulence, and they considered to select the number of Fourier components (*k*) as seven.

## Experimental setup and measurements

The dataset used in the present study to test the P-SAT framework, has been obtained from the experimental study carried out by Deshpande and Kumar^24^. Experiments were performed in a glass-walled tilting flume of dimensions 20 m X 1 m X 0.72 m (length X width X depth). A schematic diagram of the flume has been provided in Fig. 1.

An upstream collection tank of dimensions 2.8 m X 1.5 m X 1.5 m (length X width X depth) was provided with a couple of wooden baffles installed in it to quieten the flow before entering the channel. Uniform river sand of median diameter $d_{50}$ = 1.1 mm was used as bed material in the experiments. Table 3 provides information on various physical characteristics of bed material and experimental parameters.

**Table 3:** Details of various experimental parameters.

S. no.	Bed material	$d_{50}$ (mm)	Angle of repose (dry sand), ${{\varphi }^{o}}$	Gradation coefficient, ${{\sigma }_{g}}$ (Marsh et al. 2004)	Flow depth, *h* (m)	Discharge, *Q* (m$^3$/s)	Reynolds number (*Re*) of flow
1	River sand	1.1	31.154	1.03	0.135	0.0205	31418.26

### Velocity measurements

Instantaneous velocity readings along the vertical plane were taken using a four-beam, down-looking, Vectrino+ acoustic Doppler velocimeter (ADV) probe manufactured by Nortek. The instrument collects data in a cylindrical remote sampling volume located at 5 cm below the central transmitter. The height of the sampling volume was set at 1 mm when measurements were taken very near the bed such that the sampling volume did not touch the particles on the bed surface, and at 4 mm when measurements were taken away from the bed. Data were collected at the center line of the channel cross-section at a distance of 8 m from the downstream end of the flume to minimize the effects of flow entrance and exit conditions on the measurement location. Measurements were carried out on multiple heights, and at each height, 30,000 samples were collected for 5 min (i.e., sampling rate of 100 Hz).

**Figure 2:**
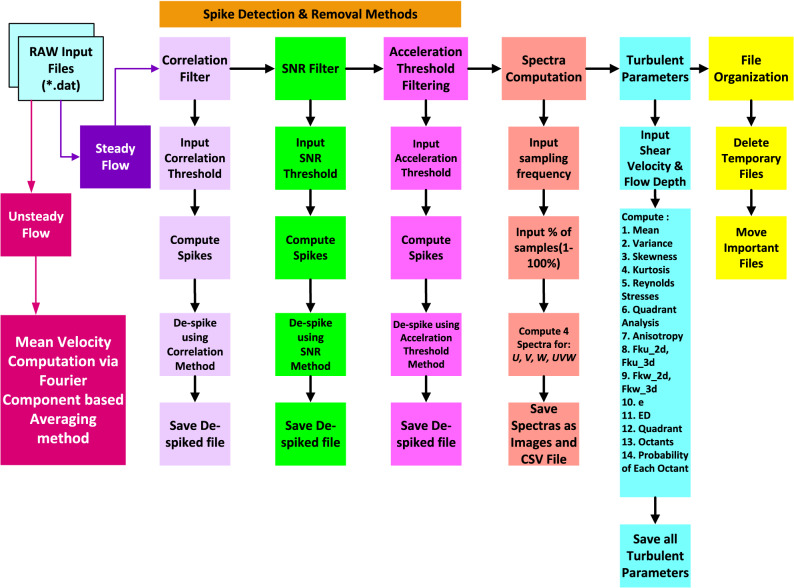
Flow diagram of the P-SAT framework.

## P-SAT configuration, usage, features, performance and limitations

### Flow diagram of the P-SAT framework

The P-SAT framework has been designed for working with steady as well as unsteady flows. Figure 2 shows the flow diagram of the P-SAT framework. The complete flow can be divided into the following main components.

- **Steady flow analysis**
  1. **Spike detection and removal methods** It consists of three Spike Detection and Removal Methods: (a) Velocity correlation filter, (b) Signal-to-Noise Ratio (SNR) filter, (c) Acceleration thresholding method^16^. The P-SAT framework de-spikes the contaminated data points obtained from the ADV and replaces them via interpolation between ends of spike method. Each of the above filtering method, requires taking an input threshold from the user for filtering purposes (a default value is also stored in the program in case the user does not wish to specify or is unsure, for example, the default value of Correlation Filter is set as 70). Once the input threshold is set, the P-SAT framework scans for the ***.dat*** files and processes them one by one. For each filtering method, it computes the spikes and replaces them by interpolation between the ends of spikes. The algorithm for the above three filtering methods along with replacements algorithm is explained in Sect. 4.4. After running each filtering method, the relevant output files are saved into ***.csv*** format by the P-SAT framework.
  2. **Turbulent statistics** The filtering methods mentioned earlier, de-spikes the noisy raw signal and converts it into a clean signal. The filtered signal is then further used for the calculations of various turbulent statistical parameters such as: time-averaged 3D velocities, velocity variances, skewness, kurtosis, Reynolds shear stresses (RSS), third order correlations, 2D as well as 3D fluxes of the turbulent kinetic energy, turbulent kinetic energy dissipation, conditional statistics of quadrants, computation of octants^25^ and their corresponding probabilities of occurrence. All these are also saved for each input file and upon every execution, these parameters are appended in the file, so that previous computation are intact with the user.
  3. **Computation of the spectral density functions of velocities** This component is used for the computation of the spectral density functions for the given input velocities. Since the ADV provides 3D velocities, the P-SAT framework computes the spectra for the velocities in streamwise, lateral, and vertical directions. A final spectra that combines all the above spectra is also plotted for user convenience. The P-SAT framework saves both the data and visualization in individual files for future reference.
  4. **File organization** The P-SAT framework creates ‘14’ files for every input ***.dat*** file it reads (for steady flow data). In order to ensure that it does not clutter the workspace of the user, the P-SAT framework creates a folder and organizes all the saved file and appends timestamp to all files. This is convenient from the user point of view as the user can perform further computation on the saved files. It also deletes any temporary files that are created during the program execution.
- **Unsteady flows**
  1. **Determination of the mean velocity component** As has been discussed in Sect. 2.2, several methods are available for the calculation of the mean velocity component from the instantaneous velocities in unsteady flow environments^22^. The P-SAT framework computes mean velocities using the Fourier-component based method, which has been found most suitable for the determination of the mean velocity component in unsteady flows^23^. The P-SAT framework can work with multiple files of unsteady flows specified in the $``input\_ensemble\_files.txt''$. This file contains list of all those files that contain unsteady flow data. For each of the file, the P-SAT framework asks for the number of components and computes the mean velocity computation via Fourier Component based Averaging method. The user has to provide a ***.csv*** file having 4 columns: *t*, *u*, *v*, *w* representing time and the 3D velocities.

### Importing data from raw files

- Input files (*.dat) : P-SAT framework requires that the input should be in ***.dat*** format and the data should be *comma* separated. This restriction has been kept since it ensures that the large code is easily managed. There are various tools available that can help to convert a space/tab separated file(s) into a comma separated format. Table 4 shows a sample ***.dat*** file obtained from the Vectrino+ software.
  When the raw velocity data is collected in any environmental flow experiment using a Nortek made Vectrino+ ADV and the Vectrino+ software, a *.vno* file is generated. This *.vno* file is further processed by the Vectrino+ software and five more files are created with *.adv*, ***.dat***, *.hdr*, *.pck*, and *.ssl* extensions. The ***.dat*** files are standard ASCII files that contain velocity time series data obtained from Vectrino+ ADV. Typically the ***.dat*** files obtained from this software consists of the data as shown in Table 4. The columns as explained below:
  - **Time** Contains the time information, depending on the sampling frequency. In this case the sampling frequency was 100 Hz and so the time interval changes every $\frac{1}{100}$ s.
  - **SL** Routine serial counter from 1 till n.
  - **Counter** A value provided by Vectrino software. It is not required by the P-SAT framework.
  - $U_i$, $V_i$, $W_i$, $W1_i$ : $U_i$, $V_i$, $W_i$ represent the three dimensional velocity data collected by the ADV. $W1_i$ is a redundant component of *W* and is not required for the current study. These velocities are raw signal (contains spikes) that need filtering methods to de-spike the noisy data.
  - **AMP**-$U_i$, **AMP**-$V_i$, **AMP**-$W_i$, **AMP**-$W1_i$ : The signal amplitude of the $U_i$, $V_i$, $W_i$, $W1_i$ as measured by the Vectrino+ ADV instrument. These values are not used by the P-SAT framework.
  - **SNR**-$U_i$, **SNR**-$V_i$, **SNR**-$W_i$, **SNR**-$W1_i$ : The SNR values of the $U_i$, $V_i$, $W_i$, $W1_i$ as measured by the Vectrino+ ADV instrument. These values are used for the SNR filtering method.
  - **Corr**-$U_i$, **Corr**-$V_i$, **Corr**-$W_i$, **Corr**-$W1_i$ : The correlation values of the $U_i$, $V_i$, $W_i$, $W1_i$ as measured by the Vectrino+ ADV instrument. These values are used for the correlation filtering method.
  It is important for the P-SAT framework that the columns of the input ***.dat*** files must be exactly in the same order for the successful execution.

**Table 4:** An instance of the *.dat* file obtained from Vectrino software.

Time (s)	SL	counter	*U* (m/s)	*V* (m/s)	*W* (m/s)	*W*1 (m/s)	AMP-*U*	AMP-*V*	AMP-*W*	AMP-*W*1	SNR-*U*	SNR-*V*	SNR-*W*	SNR-*W*1	Corr-*U*	Corr-*V*	Corr-*W*	Corr-*W*1
0	1	100011	0.2525	$-$0.0081	0.0003	$-$0.0226	108	124	131	106	19.1	20.3	21.5	19.7	44	71	73	58
0.01	2	100011	0.3524	$-$0.0602	0.0212	0.0163	146	176	171	138	21.7	23.3	23.8	22	82	88	98	93

### Dataset processing

A key component of the P-SAT framework is that it uses the Numpy arrays that loads the ***.dat*** file efficiently into the memory. The Numpy module enables us to easily import specific columns from the input ***.dat*** files and process them efficiently. After the raw ***.dat*** files are filtered, the P-SAT framework also converts them into usable *Microsoft Excel* file format. This feature was added, if the user wants to do some analysis on the filtered data using *Microsoft Excel*. We used ***.xls*** format so that we can maintain compatibility with the older version of *Microsoft Excel*. The final turbulent statistics are exported in a ***.csv*** format. The ***.csv*** format can be opened in any text editor or in *Microsoft Excel* for plotting various visualizations. Keeping ***.csv*** format enables the scientific community to write their own codes for further manipulations.

### De-spiking and replacement algorithms

The P-SAT framework implements three de-spiking method: (a) Correlation Filter (shown in Algorithm 1), (b) SNR Filter (shown in Algorithm 2), (c) Acceleration Thresholding Filtering (shown in Algorithm 3). 
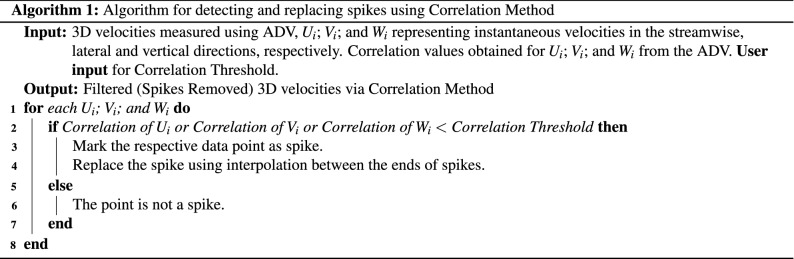


We will explain the algorithm for detecting spikes using **Correlation method**. The others filtering methods can be explained similarly. The input for Correlation Method Algorithm are the 3D velocities measured using ADV, $U_i$; $V_i$; and $W_i$ represents instantaneous velocities in the streamwise, lateral and vertical directions, respectively, and their corresponding correlation values (**Corr**-$U_i$, **Corr**-$V_i$, **Corr**-$W_i$). For every row (Line 1, Algorithm 1) that is read by the P-SAT framework (a sample row consists of the data shown in Table 4), it checks if **any** of the correlation values for $U_i$; $V_i$; and $W_i$ is less than the *Correlation Threshold* (Line 2, Algorithm 1), the corresponding velocity point is marked as spike (Line 3, Algorithm 1). The P-SAT framework provides flexibility: it provides default values for correlation threshold and also provides an option to allow the end user to enter a custom value for correlation threshold. The spike is replaced by the interpolation between the ends of spike (Line 4, Algorithm 1). The replacement algorithm is depicted in Algorithm 4. If the correlation of all the velocities are above the correlation threshold, the velocities are untouched (as they are not spike) and the next row is processed (Line 6, Algorithm 1).

The other two filtering methods are described below. 
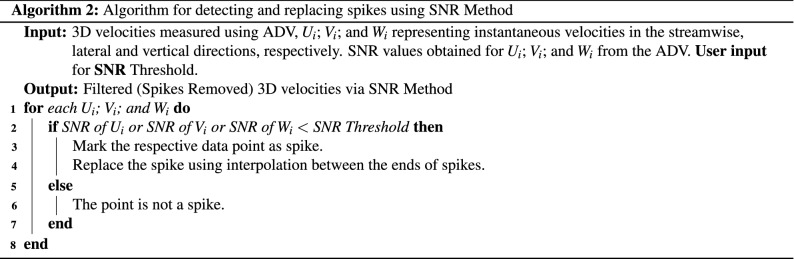


**SNR filter** Similar to the correlation filter but uses SNR (Signal-to-Noise ratio) values (using values of **SNR**-$U_i$, **SNR-**$V_i$, **SNR**-$W_i$ as explained earlier). 
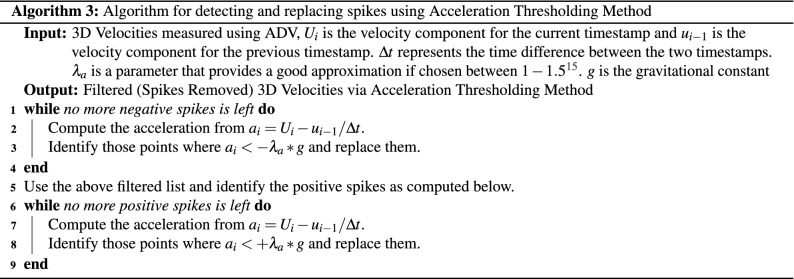

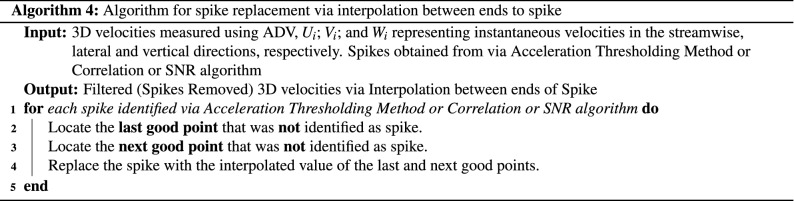


**Acceleration thresholding method** We have used the method as proposed in Goring and Nikora^16^. The acceleration thresholding method first detects the spikes and then replaces them in two phases. First phase detection is done for the negative accelerations (Lines 1–4, Algorithm 3), while the second phase detection is performed on positive accelerations (Lines 6–8, Algorithm 3). In each of the phase, there are multiple passes made through the raw data until all the data points of the given sample conform to the acceleration criteria. General formula for acceleration is $a_i = \frac{{U_i - u_{i-1}}}{ \Delta t}$. For negative accelerations all those points where $a_i < - \lambda _a g$, are marked as spike and replaced by linear interpolation between the ends of spike. The process is repeated until no more negative spikes are left. The same procedure is repeated for the positive accelerations. However, the check for positive spikes is $a_i > \lambda _a g$. The replacement strategy continues to be the same as that applied for the negative spikes (linear interpolation between the ends of spikes). The criteria is $\lambda _a g$, where $\lambda _a$ is a user defined value and *g* is the universal gravitational constant. Goring and Nikora^16^ mention that values for $\lambda _a$ should be kept between 1 and 1.5.

**Figure 3:**
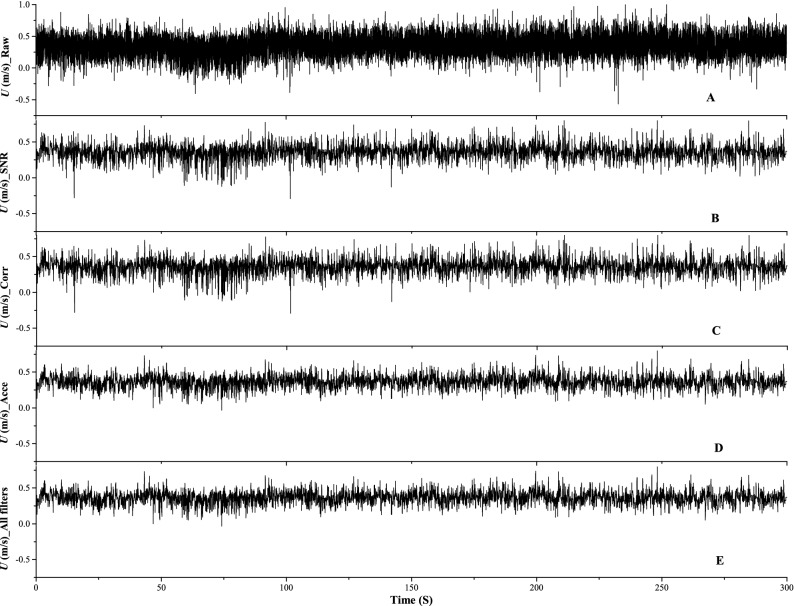
(**A**) Raw velocity signal, (**B**) Signal filtered with SNR method only, (**C**) Signal filtered with correlation method only, (**D**) Signal filtered with acceleration thresholding method only, (**E**) Signal filtered using all the methods mentioned above.

Finally, the spike replacement used is linear interpolation between ends of spike and is shown below. Given a spike data point, the algorithm takes the **last good point** that was **not** identified as spike and the **next good point** that was **not** identified as spike and performs spike replacement via linear interpolation between **last good point** and the **next good point**.

Figure 3 shows the raw velocity signal captured by the ADV and filtered signals by the P-SAT framework in a stepwise manner. The raw velocity signal at a perticular measurement location has been depicted in Fig. 3A. Whereas, the raw signal filtered by using the SNR method, velocity correlation method, acceleration thresholding method, and by the combination of all the methods mentioned above are shown in Fig. 3B–E, respectively.

**Figure 4:**
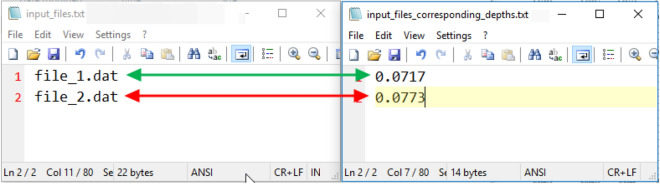
Mapping the filenames of *input_files.txt* with their corresponding depths in *input_files_corresponding_depths.txt*. We see that *file_1.dat* has a depth of 0.0717 and *file_2.dat* has a depth of 0.0773. The user can place any number of files in *input_files.txt* but should map the corresponding depths in *input_files_corresponding_depths.txt*.

### Sample code execution

For testing the P-SAT framework, we took the raw ***.dat*** files generated by the Vectrino+ software. A sample row from the raw file is displayed is in Table 4. For user convenience we have presented the sample code which is ready for the execution. After downloading the entire GitHub repository (https://github.com/mayank265/flume.git), the user just needs to extract the zip archive (Password: “PoTs_Turbulence” (password does not contain quotes)) execute $python3\ pots\_module.py$. We have taken two sample ***.dat*** files and have shown the execution of the P-SAT framework. All ***.dat*** files that need to be processed must be in the same folder where the $pots\_module.py$ is placed. Also the *input_files.txt* and *input_files_corresponding_depths.txt* should be in same folder and should be correctly mapped (see Fig. 4). The P-SAT framework creates a **Logs ** folder that contains all the errors that were recorded during the execution of the P-SAT framework. This can help for the debugging purposes.

### External libraries

The P-SAT framework requires the following libraries for the successful execution.

- ***.csv***^26^ Useful for writing files into ***.csv*** format. The final analysis is written into a ***.csv*** file.
- **datetime** Computes datetime for generation of various time format. In order to prevent conflicting filenames, we append a timestamp format to every file that is created.
- **glob** Organizing files and folders using regular expression patterns.
- **numpy**^27^ The core library for manipulating the ***.dat*** files and applying various operations on them. Numpy processes the ***.dat*** files efficiently by converting them to numpy arrays.
- **scipy** Provides libraries for various statistical parameters like kurtosis, skewness etc.
- **os** All the filtered files are stored in a “Filtered_Timestamp” folder which is generated by the os module.
- **timeit** For computing the code execution time.
- **xlwt**^28^ For converting ***.dat*** files into *Microsoft Excel* format ***.xls***.

The user can use $pip\ install\ package\_name$ to install the libraries. The authors would recommend installing the Anaconda^29^ environment which is cross platform package and installs all the above packages with the inbuilt installer.

**Figure 5:**
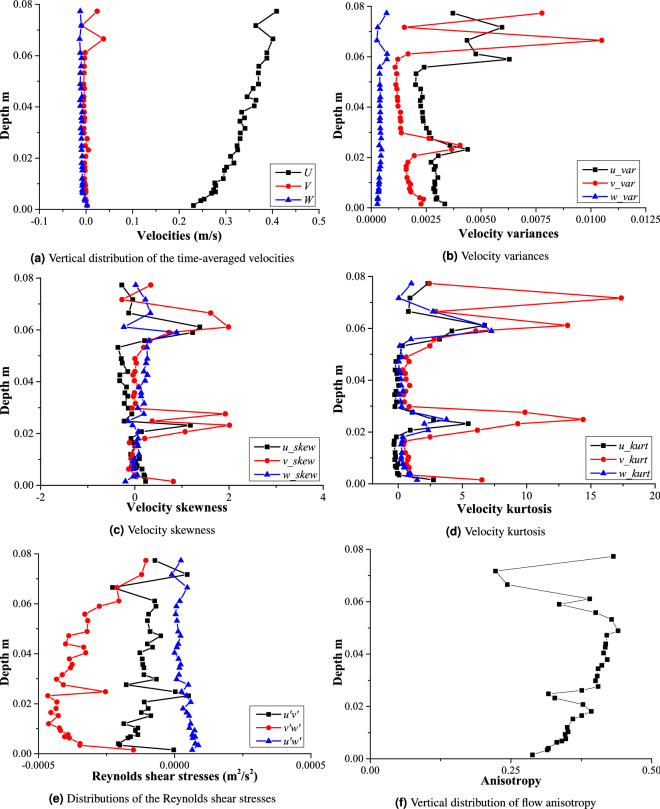
Visualizations that can be derived from the P-SAT framework.

**Figure 6:**
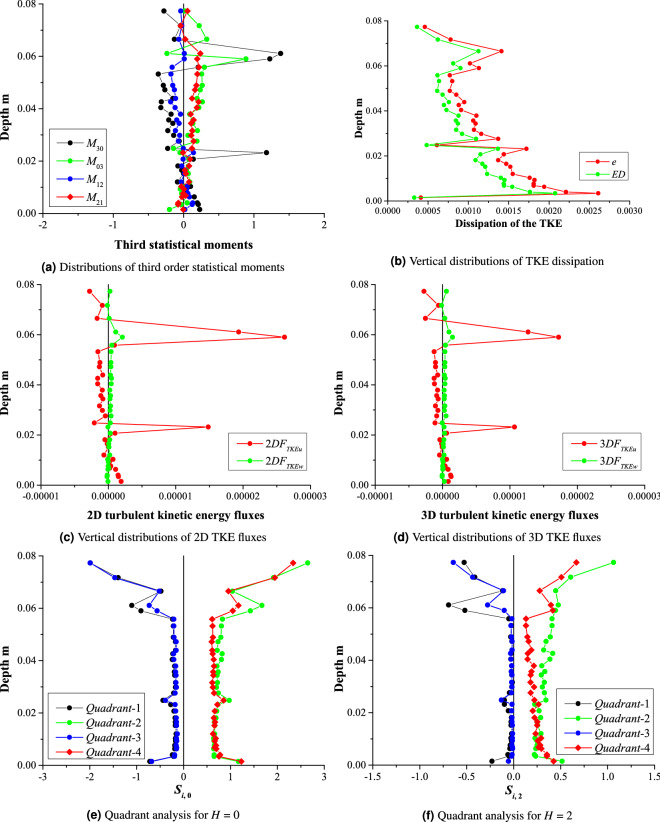
Visualizations that can be derived from the P-SAT framework (Continued...).

**Figure 7:**
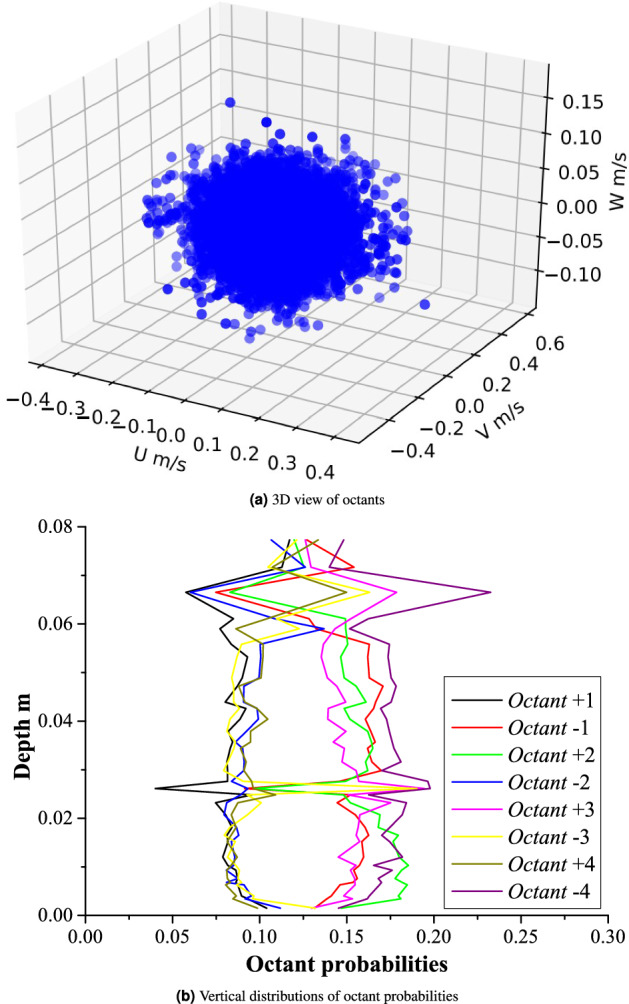
Visualizations that can be derived from the P-SAT framework (Continued...).

**Figure 8:**
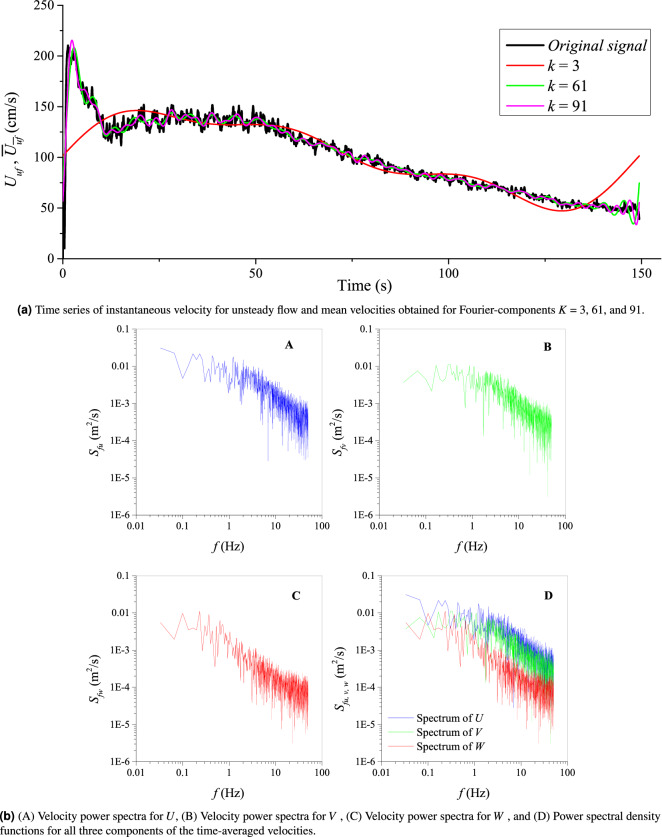
Visualizations that can be derived from the P-SAT framework (Continued...).

### Performance

The P-SAT framework is an open source module written in Python 3+ and is tested both on *Linux* and *Windows * platforms. We have used few common libraries that are easily available. Python packages are easy to install using the standard Python package installer *pip*.

We tested the performance on the following system: OS Name: Microsoft Windows 10 Pro, OS Version: 10.0.17763 Build 17763, System Type: x64-based PC, Processor: Intel(R) Core(TM) i5-6200U CPU @ 2.30 GHz, RAM: 12 GB DDR4. We have executed the P-SAT framework and provided it with a set of 33 ***.dat*** files with their respective depths. The statistical parameters of turbulence obtained via P-SAT for these 33 files are shown in Tables 5, 6, 7, 8, 9 and  10. The table header is kept self-explanatory for easier understanding. Due to space constraint, the statistical parameters of turbulence obtained via P-SAT are split across multiple tables. However, the P-SAT framework stores all the statistical parameters of turbulence in a single file.

Experimental conditions are given as follows: The velocities were measured in an open channel flow experiment at the sampling rate of 100 Hz using the Nortek made Vectrino+ ADV. The ADV was suspended from a position in the flow in order to get the 3D velocities. The $U_i, V_i, W_i,$ represent the instantaneous velocities in the streamwise ($U_i$), lateral ($V_i$), and vertical ($W_i$) directions, respectively. Each experiment was run for a period of 300 s. So in a given run, the total sample velocities obtained are $100 * 300 = 30,000$. For 33 measurements the total number of sample readings we obtained is $30000 * 33 = 990,000$ values. The P-SAT framework takes each input ***.dat*** file as input, does the pre-processing, then applies three filters to de-noise the velocity time series data and then calculates various turbulent parameters. During this process the P-SAT framework generates ***.xls*** files for all input files, the files obtained after each filtering, and a final analysis file (Parameters.csv) having the turbulent characteristics of all the input files.

The execution time for the code was 12 min 22 s. The memory usage varied between 30 MB $-$ 160 MB in a single execution. Considering the various parameters the P-SAT framework calculates, and the conversion it does, the time taken can be justified. During different runs the time taken may reduce or increase depending on the quality of the raw file. As seen in the acceleration thresholding method, the process needs to be repeated till all the spikes are removed. A noisy velocity time series data may have a large number of spikes taking more time, while a sample having few spikes is executed quickly. So, we can conclude that the P-SAT framework does not hog system resources and can be run even on a basic desktop machine.

### Tables and visualizations from the P-SAT framework

As mentioned already, the P-SAT framework generates a rich set of turbulent statistics like mean velocities, variance, skewness, kurtosis, Reynolds stresses, third order correlations, 2D as well as 3D fluxes of turbulent kinetic energy, turbulent kinetic energy dissipation, conditional statistics of quadrants, computation of Octants and their corresponding probability. Based on the rich statistics obtained, we plotted few visualizations as shown below.

Tables 5, 6, 7, 8, 9 and  10 show all the turbulent statistics generated by the P-SAT framework. We have split the tables into multiple pages as all the turbulent statistics generated by the P-SAT framework cannot be displayed using a single table.

The visualization of the turbulent statistics shown in Tables 5, 6, 7, 8, 9 and  10 is depicted in Figs. 5a, 6, 7 and 8b. Time-averaged velocities in all three directions (*U*, *V*,  and *W*) are plotted against flow depth in Fig. 5a. Vertical distributions of three components of velocity variances are shown in Fig. 5b. Fig. 5c, d depict the vertical distribution of velocity skewness, and velocity kurtosis, respectively. Reynolds shear stresses (off diagonal components of a symmetric second order tensor) are plotted against the flow depth in Fig. 5e. Vertical distribution of the flow anisotropy is shown in Fig. 5f. Figure 6a shows the distribution of the third statistical moments ($M_{30}, M_{03}, M_{12},$ and $M_{21}$) plotted against the flow depth. Vertical distributions of the turbulent kinetic energy dissipation (*e*) and its non-dimensional form (*ED*) are shown in Fig. 6b. 2D as well as 3D fluxes of the turbulent kinetic energy are plotted in Fig. 6c and d, respectively. Fractional contribution to the total Reynolds shear stress from different quadrants (quadrant analyses) for hole sizes (*H*) = 0, and 2 are presented in Fig. 6e and f, respectively. The 3D view of octants is shown in Fig. 7a, and vertical distributions of the octant probabilities are presented in Fig. 7b. Power spectral density functions for all three components of velocities are depicted in Fig. 8b.

P-SAT framework is also able to compute the mean velocities $\overline{(U_{uf}}$) of unsteady flows from the instantaneous velocities of the unsteady flows (${{U}_{uf}}$) by using the Fourier-component method described in the theoretical development section. Figure 8a shows an example of the time series of instantaneous velocities ${{U}_{uf}}\left( t \right)$ and the mean velocity component $\overline{{{U}_{uf}}}\left( t \right)$. The P-SAT framework allows the user to select any random number of Fourier components. It can be observed from Fig. 8a that the mean velocity component comes very close to the instantaneous velocities for the number of Fourier components (*k*) at ninety one.

**Table 5:** Statistical parameters of turbulence obtained via P-SAT.

Filename	Depth	*U*	*V*	*W*	$u\_var$	$v\_var$	$w\_var$	$u\_skew$	$v\_skew$	$w\_skew$	$u\_kurt$	$v\_kurt$	$w\_kurt$
File1	0.0773	0.40903	0.02397	$-$0.01251	0.00372	0.00777	0.0007	$-$0.27849	0.33847	0.01724	2.31085	2.44263	1.02965
File2	0.0717	0.36454	$-$0.00931	$-$0.01033	0.00595	0.00151	0.0003	$-$0.04423	$-$0.27285	0.22283	0.92397	17.36672	0.0482
..	..	..	..	..	..	..	..	..	..	..	..	..	..
File33	0.0015	0.2306	0.00225	0.00204	0.00335	0.00227	0.00028	0.2295	0.81738	$-$0.20091	2.75305	6.51751	1.47359

**Table 6:** Statistical parameters of turbulence obtained via P-SAT (Continued...).

Filename	Depth	$u\_stdev$	$v\_stdev$	$w\_stdev$	$\overline{u'v'}$	$\overline{u'w'}$	$\overline{v'w'}$	Anisotropy	$M_{30}$	$M_{03}$	$M_{12}$	$M_{21}$
File1	0.0773	0.06101	0.08817	0.02637	$-$7.24E-05	$-$0.000105663	2.27E-05	0.43212	$-$0.27848	0.01718	$-$0.0393	0.05581
File2	0.0717	0.07717	0.0388	0.01718	4.65E-05	$-$0.0001211	$-$1.02E-05	0.22264	$-$0.04428	0.22277	$-$0.02444	$-$0.04098
..	..	..	..	..	..	..	..	..	..	..	..	..
File33	0.0015	0.05789	0.04767	0.01669	$-$3.66E-06	$-$0.000151116	6.45E-05	0.28823	0.22944	$-$0.20087	0.01918	$-$0.00581

**Table 7:** Statistical parameters of turbulence obtained via P-SAT (Continued...).

Filename	Depth	$2Df_{TKEu}$	$2DF_{TKEu}$	$2Df_{TKEw}$	$2DF_{TKEw}$	$3Df_{TKEu}$	$3DF_{TKEu}$	$3Df_{TKEw}$	$3DF_{TKEw}$	TKE
File1	0.0773	$-$4.87E-05	$-$2.77E-06	4.34E-06	2.47E-07	$-$4.86E-05	$-$2.77E-06	1.01E-05	5.77E-07	0.006095886
File2	0.0717	$-$1.57E-05	$-$8.92E-07	$-$2.30E-06	$-$1.31E-07	$-$1.05E-05	$-$5.95E-07	$-$2.63E-06	$-$1.49E-07	0.003877757
..	..	..	..	..	..	..	..	..	..	..
File33	0.0015	3.36E-05	1.91E-06	$-$9.44E-07	$-$5.37E-08	1.49E-05	8.50E-07	4.76E-06	2.71E-07	0.002951285

**Table 8:** Statistical parameters of turbulence obtained via P-SAT (Continued...).

Filename	Depth	$S_{1,0}$	$S_{2,0}$	$S_{3,0}$	$S_{4,0}$	$S_{1,2}$	$S_{2,2}$	$S_{3,2}$	$S_{4,2}$	*e*	ED
File1	0.0773	$-$1.98977	2.64558	$-$1.99061	2.3348	$-$0.52656	1.06369	$-$0.64186	0.66729	0.00046	0.00036343
File2	0.0717	$-$1.39548	1.91653	$-$1.46716	1.94611	$-$0.41369	0.60727	$-$0.43675	0.50796	0.00078	0.000621967
..	..	..	..	..	..	..	..	..	..	..	..
File33	0.0015	$-$0.72003	1.16876	$-$0.67938	1.23064	$-$0.23043	0.51603	$-$0.05579	0.42531	0.00041	0.000330071

**Table 9:** Statistical parameters of turbulence obtained via P-SAT (Continued...).

Filename	Depth	Octant+1	Octant-1	Octant+2	Octant-2	Octant+3	Octant-3	Octant+4	Octant-4	Total Sample
File1	0.0773	3516	3813	3592	3200	3784	3639	4005	4445	29994
File2	0.0717	3383	4623	3763	3786	3883	3143	3216	4197	29994
..	..	..	..	..	..	..	..	..	..	..
File33	0.0015	3121	3944	4404	3358	3889	3956	2963	4358	29993

**Table 10:** Statistical parameters of turbulence obtained via P-SAT (Continued...).

Filename	Depth	Probability Octant+1	Probability Octant-1	Probability Octant+2	Probability Octant-2	Probability Octant+3	Probability Octant-3	Probability Octant+4	Probability Octant-4	Min Octant Count	Max Octant Count
File1	0.0773	0.11722	0.12713	0.11976	0.10669	0.12616	0.12132	0.13353	0.1482	3200	4445
File2	0.0717	0.11279	0.15413	0.12546	0.12623	0.12946	0.10479	0.10722	0.13993	3143	4623
..	..	..	..	..	..	..	..	..	..	..	..
File33	0.0015	0.10406	0.1315	0.14683	0.11196	0.12966	0.1319	0.09879	0.1453	2963	4404

### Limitations and precautions to be taken while using the P-SAT module

Following points needs to be taken care by a user executing the P-SAT framework.

1. The P-SAT framework requires a strict file naming convention for the input ***.dat*** input files. It is preferred that the input files should not have spaces or any special charter except “_”.
2. Ensure all source files are strictly *comma* separated files. That implies that all source files have values separated by ‘,’.
3. The *input_files.txt* and *input_files_corresponding_depths.txt* are correctly mapped (see Fig. 4).
4. The libraries mentioned in Sect. 4.6 must be installed before running P-SAT framework.
5. Ensure that no file/filename is changed while the P-SAT framework is executing. It may result in an erroneous output.

## Discussion

There are various commercial tools as well as free tools available for calculation of the turbulent parameters. Commercial software provide users with a simple Graphical User Interface (GUI) and allows them to filter the raw velocity time series data, but they are usually expensive. If we check out the formulas provided in Sect. 2, it can be seen that few of them can be computed in *Microsoft Excel* while for others formulas can be built. In fact the authors initially began with turbulent analysis on *Microsoft Excel* as it provided a lot more flexibility in terms of plotting graphs and was helpful for analysis. However, the authors faced the following issues while working with *Microsoft Excel*: (a) For Quadrant and Octant analyses, the formulation in *Microsoft Excel* was tedious. (b) Also, an oversight in one of the formula can hamper the results of all subsequent formulas. (c) With the data size growing (as in our case, nearly $\approx$ 1 million values over 33 files), the computations began to get heavy resulting in *Microsoft Excel* occasionally freezing. (d) The process to merge all the statistical data after analysis was to be done manually making the whole process cumbersome. (e) Working on unsteady flows requires Fourier averaging which is challenging to compute in *Microsoft Excel* and to the best of author’s knowledge, no such tool exists that provides for the computation of unsteady flows with a user chosen values of *k* (number of components). All this took a lot of time, and there were always room for errors.

We wanted to provide the community with a free and open source module that can perform all the tasks hassle free. The P-SAT framework is completely open source, which allow the developers and scientific community to extend it to meet their needs and purposes.

## Conclusion and future work

We provide the end user with an open source P-SAT framework that can enable the user to filter the raw velocity time series data obtained from the Nortek Vectrino+ ADV and compute various turbulent parameters. We believe that P-SAT framework is a first of its kind framework that can completely automate the process of parsing, filtering, computation of various turbulent statistics, spectra computation for steady flows. For unsteady flows the P-SAT framework obtains mean velocities from instantaneous velocities by using Fourier-component based averaging method. P-SAT framework also saves all the processed data files so that the end user can use it for future purposes without actually needing to run the P-SAT framework again on the same set of files.

The authors have put the source code on GitHub and would welcome suggestion and improvements for the same. We believe that the P-SAT framework will help the scientific community working with environmental hydraulics by providing a means to easily filter and compute the parameters. As the core code is developed in Python, it runs smoothly on Windows, Linux and MAC based operating system.

Right now the P-SAT framework works on a command line based environment, in the future we would like to build a GUI module for the same. The GUI module will allow for richer user experience, however, for this paper, we restrict it to command line environment only.

## Methods

We have uploaded the required dataset and the source code for the P-SAT module on Zenedo. The url for accessing the data and code is: (https://doi.org/10.5281/zenodo.4097839). After downloading the zip file from the Zenedo repository, the user just needs to extract the zip archive and execute $python3\ pots\_module.py$.
